# Supplementary material for: Complexome profiling of the Chlamydomonas psb28 mutant reveals TEF5 as an early PSII assembly factor
Source: Plant Cell. 2025 Mar 17;37(6):koaf055. doi: 10.1093/plcell/koaf055 (PMC12164590; doi:10.1093/plcell/koaf055)
Supplement: koaf055_Supplementary_Data [file koaf055_supplementary_data.zip › psb28_interactive.zip!/plots/intensity/qualityPlots/peptides/QCR7_Cre06.g262700.t1.1.html]

### Cre06.g262700.t1.1

Trivial name: QCR7  
SeqCoverage [median]: 58.50  
Mass: 14.04 kDa  
  
 

| Sequence | MissedCleavage | Mass [Da] | wtIntensity | muIntensity | Score |
| --- | --- | --- | --- | --- | --- |
| AQLGAPAPYTR | 0 | 1143.60 | 38972.55 | 42796.91 | 152.86 |
| ERAQLGAPAPYTR | 1 | 1428.75 | 15679.29 | 13402.17 | 75.46 |
| FDDLQDPLKDEDVAEALR | 1 | 2088.00 | 24840.12 | 18974.16 | 193.22 |
| FDDLQDPLKDEDVAEALRR | 2 | 2244.10 | 8964.33 | 0.00 | 96.67 |
| LPPDVVVAR | 0 | 964.57 | 1823.77 | 3065.32 | 95.74 |
| QTPELSYLQDVLNEVR | 0 | 1902.97 | 9208.18 | 0.00 | 179.41 |
| QVALPVFNSLATTYR | 0 | 1678.90 | 4314.02 | 0.00 | 152.96 |
| RLPPDVVVAR | 1 | 1120.67 | 0.00 | 581.84 | 41.54 |
|  |  |  | 103802.3 | 78820.4 |  |
